# 3D in vitro hydrogel models to study the human lung extracellular matrix and fibroblast function

**DOI:** 10.1186/s12931-023-02548-6

**Published:** 2023-10-05

**Authors:** Sakshi Phogat, Fama Thiam, Safiya Al Yazeedi, Filsan Ahmed Abokor, Emmanuel Twumasi Osei

**Affiliations:** 1 Department of Biology, Okanagan Campus, University of British Columbia, 3187 University Way, ASC366, Kelowna, BC V1V1V7 Canada; 2 Centre for Heart Lung Innovation, St. Paul’s Hospital, Vancouver, BC V6Z 1Y6 Canada

**Keywords:** Lung fibroblasts, 3D hydrogels, Extracellular matrix (ECM), Fibrosis, ECM stiffness

## Abstract

The pulmonary extracellular matrix (ECM) is a macromolecular structure that provides mechanical support, stability and elastic recoil for different pulmonary cells including the lung fibroblasts. The ECM plays an important role in lung development, remodeling, repair, and the maintenance of tissue homeostasis. Biomechanical and biochemical signals produced by the ECM regulate the phenotype and function of various cells including fibroblasts in the lungs. Fibroblasts are important lung structural cells responsible for the production and repair of different ECM proteins (e.g., collagen and fibronectin). During lung injury and in chronic lung diseases such as asthma, idiopathic pulmonary fibrosis (IPF) and chronic obstructive pulmonary disease (COPD), an abnormal feedback between fibroblasts and the altered ECM disrupts tissue homeostasis and leads to a vicious cycle of fibrotic changes resulting in tissue remodeling. In line with this, using 3D hydrogel culture models with embedded lung fibroblasts have enabled the assessment of the various mechanisms involved in driving defective (fibrotic) fibroblast function in the lung’s 3D ECM environment. In this review, we provide a summary of various studies that used these 3D hydrogel models to assess the regulation of the ECM on lung fibroblast phenotype and function in altered lung ECM homeostasis in health and in chronic respiratory disease.

## Background

The lung mesenchyme is an extensively organized mesodermal tissue that incorporates cells in a complex intertwined extracellular matrix (ECM) protein fiber network [1, 2]. Previously regarded as non-stimulatory tissue containing material only significant for providing structural support and stability, the lung-ECM is now recognized for playing a bioactive role in various physiological and pathological processes [3]. One of the main structural cells in the lung ECM are the fibroblasts which are found in both the lung airways and alveoli [4]. Irrespective of their location, lung fibroblasts are essentially linked to connective tissue fibers and have specialized invaginations and protrusions that assist in their interaction with other cells and surrounding ECM structures [4–9]. Fibroblasts are the main producers of the glycoprotein and proteoglycan rich ECM and are also essential for organizing the various protein fibers that make up the ECM [10–12]. The lung ECM in turn supports the phenotype and function of fibroblasts and other cells via biochemical and micro-architectural cues that create signaling niches and provide positional instructions [13]. These lung fibroblast-ECM interactions are essential for reconstructing functional tissue, during various stages of lung inflammation and ECM remodeling in tissue homeostasis but have been shown to become abnormal in various lung diseases [10, 13, 14].

Modifications in the airway or alveolar ECM have been identified in numerous pathological profiles of different respiratory diseases [3, 15, 16]. Pathological changes in the ECM (e.g., molecular composition and intrinsic stiffness) during lung injury or disease plays an active role in modulating cellular behavior, especially considering the reparative function of fibroblasts [2, 4]. As there are no drugs that can effectively reverse fibrosis, the role of the ECM in orchestrating cellular (mainly fibroblast) responses in multiple lung diseases that further increases the production and deposition of ECM proteins to drive fibrosis and disease progression has been the focal point of multiple therapeutic research studies [3, 4]. In most of this traditional research, the commonly used two-dimensional (2D) monolayer cell-culture systems which are simple and high throughput allow for the study of mechanisms behind increased expression of different ECM proteins by fibroblasts in different pathological conditions. However, 2D cell culture models are restrictive in capturing and recreating the complex cellular -ECM lung microenvironment. In addition to that, owing to their inability to mimic the biochemical and three-dimensional (3D) anatomical complexities of human tissues, these assays may also yield deceptive data, which is then used to inform subsequent animal studies [17, 18]. Further to this bottleneck, the well documented lack of similarities between animal models and the human anatomy and physiology ensures that most preclinical findings are not translatable to the in vivo environment [19]. Therefore, to mimic the complex 3D relationship between the pulmonary ECM and lung fibroblasts, it has been essential to establish 3D hydrogel models in which cells are embedded in configurations that mimic the temporal, spatial and cell type specific connections found in the in vivo lung environment [20]. These 3D hydrogel models are in vitro tissue constructs which form part of the current state-of-the-art biomimetic technology that are able to mimic the structural and functional features of real tissue such as complex cellular interactions and crosstalk within the lung ECM [21, 22]. This is because cells embedded in 3D hydrogels are enabled in their ability to interact with each other and the surrounding ECM in all directions much like in tissue and unlike in 2D culture [21–24]. The advancements in using 3D hydrogel models to recapitulate lung fibroblast-ECM relationships have enabled the assessment of the effect of the lung’s 3D ECM microenvironment on fibroblast phenotype and function in tissue homeostasis and disease [25].

In this review we seek to summarize current data in the field of lung fibroblast biology and provide an overview of how understanding the functional and mechanical relationship between lung fibroblasts and its surrounding microenvironment through the use of 3D hydrogel models, may enable the understanding of how the 3D ECM environment affects fibroblast phenotype and function and vice versa. We first provide an overview of the various types of 3D hydrogels used for lung fibroblast studies. We then summarize studies that have used 3D hydrogels to assess the influence of stiffness on lung fibroblasts before assessing idiopathic pulmonary fibrosis (IPF), chronic obstructive pulmonary disease (COPD) and asthma 3D hydrogel models and studies that are using hydrogel technologies to address lung therapeutics.

## Main text

### 3D Hydrogel culture models to study lung fibroblast phenotype

Hydrogels are crucial biomaterials for building 3D in vitro models owing to their similarities to the native extra cellular matrix (ECM) in the human body [20]. Hydrogel scaffolds are an interconnected 3D network of hydrophilic polymers (natural or synthetic) where cells for experimentation can be either encapsulated or seeded on top of their microfilaments to form a micro-gel [26, 27]. They have great ability to hold large amounts of fluid while maintaining a distinct 3D structure similar to the hydrated natural ECM [28]. Hydrogel 3D models collectively offer new routes to study and experiment with cellular mechanisms in four dimensions (i.e., x, y, z, and w) where the cellular functions are observed in the 3-dimensional space and the fourth dimension of time [21, 29]. The lung ECM possesses a complex variety of structural proteins that were traditionally thought to only provide architectural integrity and support [30]. However, through 3D hydrogels, it is now known that the lung ECM milieu fundamentally affects cellular behavior and function [21, 31]. In lung research, there are two main types of hydrogel scaffolds used; natural hydrogels and synthetic hydrogels [32, 33].

Hydrogels made from natural polymers found in the native ECM (e.g., hyaluronic acid, collagen) have prominent bioactive features that allow them to interface favorably with cells [34, 35]. Natural hydrogels can be classified into two main types, i.e., protein and polysaccharide-based hydrogels [34]. Collagen, a major structural protein of the lung ECM, is the most widely used natural polymer for building 3D hydrogel systems used for cell-embedded 3D hydrogel studies [30, 36]. Most collagen scaffolds are prepared using type I collagen and can be easily modified by cross-linking with common techniques such as temperature dependent gelation, gel-compression and other chemical methods e.g., acid-base titration and glutaraldehyde gelation [37–39]. Another commonly used natural hydrogel is Matrigel, which is composed mainly of collagen IV, glycoproteins and various growth factors found in the basement membrane protein. It is a thermo-sensitive solution that turns into a hydrogel at temperatures higher than 12 °C and is used widely for 3D cell (co-)cultures, bioprinting and tissue engineering to support independent epithelial cell culture and their co-culture with other cell types such as fibroblasts [40]. Further, gelatin methacrylate (GelMa) is another natural ECM mimicking polymer commonly used for building 3D hydrogel models [41–43]. GelMa is a protein-based polymer that is manufactured by reacting methacrylic anhydride (MA) with gelatin, a naturally occurring hydrolyzed derivative of collagen [44]. GelMa is a photo-polymer, hence it is cross-linked into a solid hydrogel with the help of a biocompatible and non-toxic photo-initiator (e.g., Lithium phenyl-2,4,6-trimethylbenzoylphosphinate (LAP)) and UV light [43, 45].

Hyaluronic acid (HA) is an important component of connective tissue and also a natural hydrogel that is attractive for the construction of 3D models [46]. HA has a unique anisotropy and can be chemically modified by radical polymerization into soft or stiff hydrogel scaffolds [47]. In addition to HA, alginate is another natural polysaccharide that is commonly used in building 3D hydrogel systems because of its rapid cross-linking property [48, 49]. It is a sea-weed derived polymer that is cross-linked by exposure to calcium chloride (CaCl_2_) solution [48–50]. Natural hydrogel scaffolds can also be prepared by sourcing the ECM through allogeneic or xenogenic lung decellularization, commonly done via perfusion of detergents or salt solutions [51, 52]. Here, the cellular content of the lung is removed leaving the intact ECM that is used for a wide range of applications such as, substrates for 3D cell culture, bioinks for bioprinting and establishing organoids as well as in therapeutics to promote tissue repair after an injury [51, 53–55]. While these ECM bio-scaffolds retain the cellular environment to allow inherent biological activity of the natural matrix, support cell growth and promote constructive tissue remodeling, they also come with challenges such as damage to the ECM structure due to the detergents used during decellularization. Modifying the stiffness of these hydrogel matrices can enable the study of how specific cells spread and sprout and how they behave in a normal vs. fibrotic ECM environment [56–58]. One of the main advantages of using a natural polymer in building a 3D hydrogel model is their dominating bio-active features such as cell-adhesion motifs, non-immunogenicity, non-inflammatory and biodegradable properties [59–61]. In addition to that, it is also easy to covalently integrate peptide ligands and cell membrane receptors to stimulate adhesion, spreading and proliferation of cells within the natural hydrogel matrix [46, 62]. However, as opposed to synthetic polymer-based hydrogels, natural polymer-based hydrogels generally have poor mechanical strength and low stability [34, 35, 63].

Comparatively, hydrogels made from synthetic hydrophilic molecules (e.g., polyethylene glycol (PEG) and polyacrylamide (PA)) offer better mechanical properties (e.g., strength and stability) resembling the native lung tissue but lack inherent biologically active features [64]. Synthetic polymers offer better control over structure (e.g., cross-linking density) and property (e.g., mechanical strength and chemical response to stimuli) that are important for recapitulating lung tissue architecture [65]. The most commonly used synthetic molecules for 3D cell cultures are PEG and PA [28, 66]. PEG is a predominantly used synthetic material when establishing 3D models due to its minimum protein adsorption, hydrophilicity and customizable cross-linking chemistry [66]. PEG is mainly used to study the changes in cell behavior with regards to matrix stiffness and fibrosis [67]. PA hydrogels, on the other hand, are popular in cell mechanical studies due to advantages such as, high-resolution cell images resulting from their transparent nature and easy customizability of their stiffness and surface functionality [68]. Synthetic polymers are however, inert and do not have any effect on cellular activity and therefore, are generally combined with bioactive material to enhance their biocompatibility (i.e., cell adhesion motifs or growth factors) [33].

To recapitulate the entirety of the complex properties observed in the *in-vivo* ECM microenvironment, it is sometimes not enough to use a single polymer hydrogel. Therefore, to fully meet the functional and structural characteristics of the 3D ECM in some tissues, a composite or hybrid hydrogel with more than one polymer possessing synergistic properties is designed [21, 69]. Composite hydrogels overcome the weak mechanical properties of natural scaffolds while providing bioactive ECM components to modulate cell behavior [70, 71]. A few of the most common examples of these are GelMa/PEG, alginate-PA, and collagen-PA hydrogels [66, 72]. Hydrogel models have successfully been used to mimic the physical and mechanical properties of the natural vs. diseased ECM and have been employed to explore how lung fibroblasts and other cells are affected by changes in their physical environment [73–75].

### 3D Hydrogel culture models assessing the role of ECM stiffness in regulating lung fibroblast phenotype and function

The pulmonary ECM is now understood to be a bioactive environment that regulates cell fate, phenotype and function, with essential roles in the maintenance of tissue homeostasis and regulation of injury-repair responses [3, 4]. Changes in the lung ECM architecture, viscoelasticity and content are associated with the pathobiology of many chronic respiratory diseases, including asthma, COPD and IPF [13, 14]. In asthma, fibrosis and remodeling is predominantly observed in the airway ECM, in COPD it is mainly observed in both the airways and parenchyma whereas in IPF it is mainly observed in the lung parenchyma [14]. Hydrogels have tunable mechanical properties that can be employed to model load-bearing lung tissue and study how changes in tissue stiffness is contributing to cellular differentiation in fibrotic regions [26, 27]. In line with this, various studies employing 3D hydrogels have been used to investigate the role of the stiff lung ECM environment in controlling the mechanisms that regulate the differentiation of lung fibroblasts to the highly synthetic myofibroblasts (fibroblast-to-myofibroblast transition (FMT)) as well as the differentiation of pulmonary epithelium to mesenchymal cells (epithelial-to-mesenchymal transition (EMT)) [76–78].

To aid in understanding the relationship between pulmonary ECM stiffness and various fibrotic responses during FMT, Marinkovic et al., improved methods for higher-throughput traction measurements which was used to study how the mechanical properties (i.e. stiffness) of the 3D matrix impact fibroblast contractility after stimulating cells with the major fibrotic mediator transforming growth factor (TGF)-β1 [79]. Here, PA hydrogels of different stiffnesses ranging from 0.3 to − 20 kPa were prepared and conjugated with fluorescent microsphere beads of different emission wavelengths. Gels were then seeded with IMR-90 lung fibroblast cell-lines, in the presence or absence of TGF-β1 treatment. With traction measurements, it was determined that lung fibroblasts exert lower forces on softer matrices as opposed to greater forces on stiffer matrices [79–82]. Moreover, exogenous TGF-β1 treatment increased the force generating capacity of lung fibroblasts and promoted α-smooth muscle actin (α-SMA) expression (phenotypic marker of FMT) on stiffer matrices (mimicking the fibrotic lung). However, these effects were not observed on softer matrix substrates mimicking the stiffness of healthy lungs, despite TGF-β1-dependent Smad2/3 activation just as in IMR90 fibroblasts on the stiffer matrix [79]. This points to an interaction between the stiff lung ECM environment and fibrotic mediators, in fibroblast force generation and FMT. Further, Liu et al., adapted a similar method and engineered a collagen I functionalized PA hydrogel system displaying a 1-dimension (1D) gradient with a shear modulus ranging from 0.1 to 50 kPa on which CCL-151 lung fibroblast cell lines were seeded [83, 84]. Interestingly, significant changes were observed in CCL-151 lung fibroblast morphology [84], which changed from attenuated round cells at lower stiffness to spindle shaped with dendrites on intermediate stiffness gels, and parallel swirls of spindle-shaped cells at higher stiffnesses [84–86]. Furthermore, a stiffness-dependent suppression of cyclooxygenase-2 (COX-2) expression and synthesis of prostaglandin E2 (PGE2) during fibrogenesis was reported in lung fibroblasts to be associated with these changes [84]. Taken together, through hydrogel model studies, it has been shown that pulmonary fibroblasts are highly sensitive to mechanical and chemical changes (stiffnesses ranging from 0.1 to 50 kPa and growth factors e.g., TGF-β1) in the lung environment and change their contractile machinery as a consequence of cell-ECM mechanohomeostasis during FMT [79, 84].

In line with EMT as a suggested pathologic mechanism in fibrotic lung diseases, a high production of mesenchymal cells derived from epithelial cells had been suggested to contribute to tissue stiffness [78]. However, Brown and colleagues contradicted this observation and showed that tissue stiffness cues precedes fibrotic responses [78, 87]. Here, experiments were designed to investigate the dependence of lung EMT on the biochemical signals produced by ECM proteins by testing how changing the lung ECM stiffness affects EMT and whether this phenomenon is reversible [88, 89]. To do this, the potential reliance of alveolar epithelial cells (AEC) on integrin αvβ6/contraction-dependent TGF-β activation during EMT on stiff fibronectin (Fn) matrices was examined [78]. Primary alveolar epithelial type II and RLE-6TN rat alveolar cells were seeded on PA gels of different stiffnesses coated with Fn or Laminin (Ln), ranging from 2 to 32 kPa for 5 days. When tested for EMT by immunofluorescence (IF) staining of actin, α-SMA, epithelial and mesenchymal markers, alveolar epithelial type II and RLE-6TN cells grown on lower-stiffness Ln matrices (2 kPa) displayed rounded epithelial morphologies whereas, on higher stiffness Fn matrices (16-32 kPa) cells were elongated, contractile and displayed aligned actin filaments similar to stress fibers [78]. Furthermore, it was observed that low levels of TGF-β caused primary alveolar epithelial type II and RLE-6TN cells to undergo EMT however, upon the removal of TGFβ, cells reverted to an epithelial phenotype [78]. The data in this study suggests that increased epithelial cell contractility on stiff (Fn-coated) matrices causes integrin-mediated TGF-β activation and EMT; and that abnormal EMT-derived mesenchymal cells have the potential to revert back to their normal phenotype if the fibrotic stimulant is reversed, which provides important insights for future therapeutic studies [78].

In the active lung mucosa, cells do not solely experience unidirectional changes in matrix stiffness rather a variety of changes that can be cyclic, continuous and reversible [90]. For instance, during lung injury and repair, the tissues undergo stiffening and softening sequences and static hydrogels, e.g., Ln or Fn coated PA gels as mentioned above might not be the best option to mimic these dynamic biological events [90–94]. Hence in 2019, Fu et al., engineered a novel protein-based hydrogel with cyclic and reversible mechanics that can be tuned in a large range of stiffnesses (6 kPa and 20 kPa) via glutathione, a reducing agent [91, 95, 96]. Here, the cell culture medium was switched between the oxidizing and reducing state in a cyclic manner to study how lung fibroblasts respond to continuously changing hydrogel mechanics [96]. Under oxidizing conditions, human lung fibroblasts (HLF) changed morphology and went from a high cell spread state to a low cell spread state, leading to a significant increase in the cell area along with a slight decrease in the cell roundness [96]. When conditions were switched back to a reducing state, the opposite was observed, along with clear visibility of actin stress fibers on HLFs indicating better adherence on stiff matrices [96–98]. This study suggests that HLFs have the ability to continuously sense hydrogel stiffnesses and produce a fully reversible and dynamic mechanoresponse by changing the cell morphology and phenotype (cell spreading, cell area, cell roundness, and presence of actin stress fibers).

**Fig. 1:**
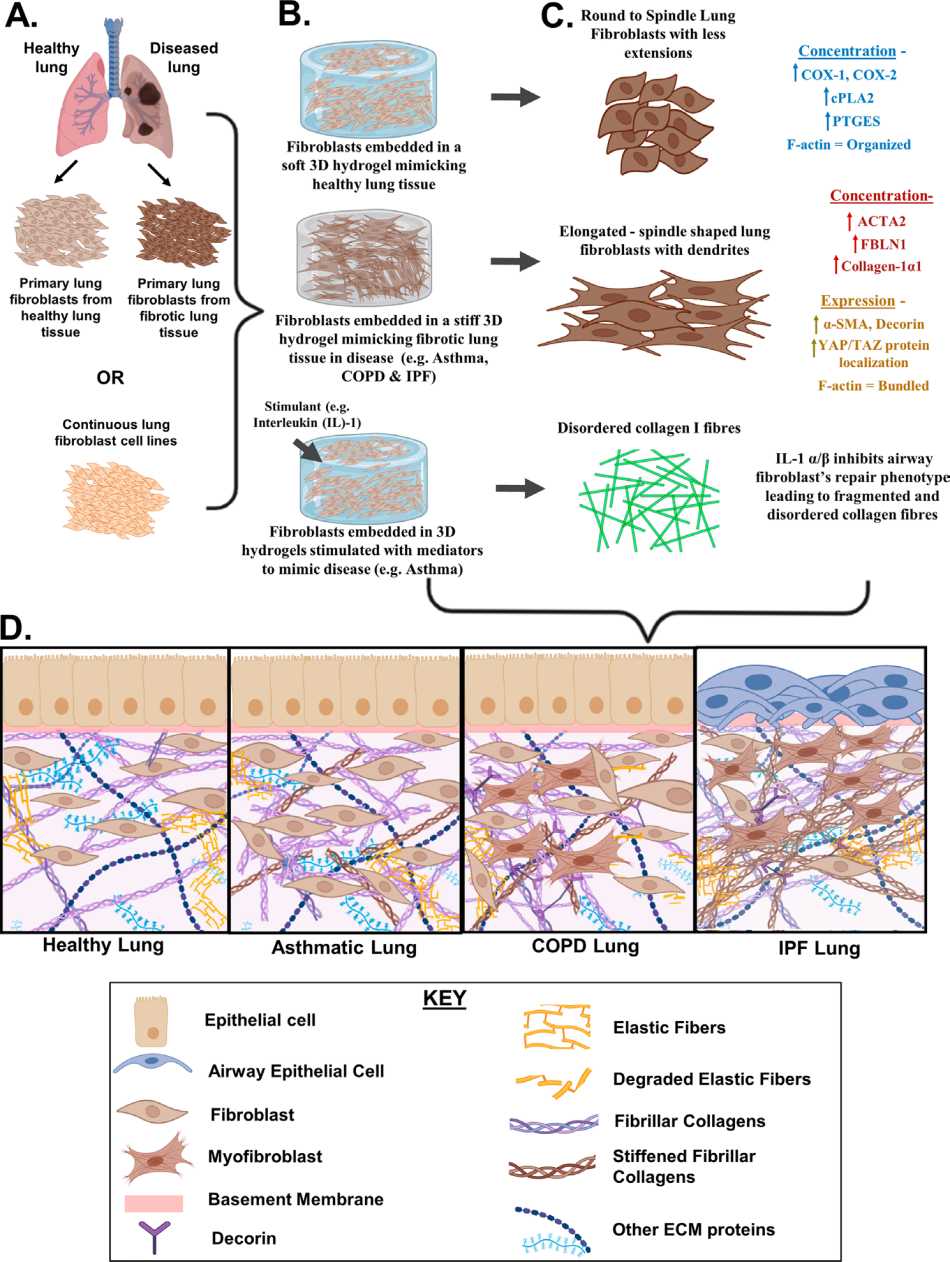
3D in vitro hydrogel culture models assessing the role of ECM in controlling lung fibroblast phenotype and function. (**A**) Human lung fibroblasts obtained from primary or continuous cell lines cultured on soft hydrogels produce a round morphology with less dendritic extensions, organized F-actin and have higher levels of antifibrotic mediators (e.g., cPLA2 COX-2, COX-1, PTGES and PGE2). **(B)** HLFs obtained from primary and continuous cell lines cultured on stiff hydrogels produce elongated morphology with dendritic extensions, bundled F-actin, and fibrotic phenotype coupled with fibroblast-to-myofibroblast transition (e.g., increased YAP/TAZ protein localization increased expression of ACTA2, COL-1A1, FBLN1 and decorin). **(C)** Mediators such as inflammatory cytokine IL-1α and IL-1β cause defective fibroblast (primary/continuous cell line and cultured in hydrogels) repair and, remodeling phenotype leading to fibrillar collagen disorganization in diseases (e.g., asthma and COPD). **(D)** Alterations in ECM composition and function in asthma COPD and IPF compared to control lungs as determined by lung-fibroblast embedded studies. In the healthy lung, the ECM consists of intact and organized elastic and collagen fibers, proteoglycans, glycosaminoglycans, etc. In the asthmatic lung, the ECM is stiff with abnormal production and disorganization of fibrillar collagens. In COPD lungs, the ECM is stiff with degraded elastic fibers, increased decorin deposition, disorganized and abnormal fibrillar collagen. In IPF lungs, there is fibrosis and remodeling in the lung parenchyma with abnormal ECM production and increased myofibroblast differentiation

All together, these studies suggest that with the help of 3D hydrogels, it is clear that lung fibroblasts are highly sensitive to stiffness changes in the native cellular environment and adapt their contractile machinery to respond accordingly(Fig. 1A &B) [90]. As a consequence of cell-ECM mechanohomeostasis, stiffer matrices cause lung fibroblasts to have increased fibroblast contractility, TGF-β1 signaling and α-SMA expression whereas soft matrices suppress fibroblast contractility, TGF-β1 signaling and α-SMA expression [79, 99]. The studies reviewed also showed that stiffer matrices cause and sustain lung pro-fibrotic environments by initiating FMT, EMT, and feedback cycles of increased fibroblast activation (involving TGF-β1 and α-SMA expression).

### 3D hydrogel culture models to assess lung fibroblast biology in IPF, COPD and asthma pathology

3D hydrogels have been used as in vitro models to mimic, characterize, and assess the biochemical and biomechanical effects of the 3D ECM microenvironment on the phenotype and function of lung fibroblasts and other pulmonary cells in different human lung diseases [21]. IPF, COPD and asthma are all chronic lung diseases that involve abnormal deposition of ECM resulting in fibrosis that is characterized by abnormalities in lung architecture and deficient gas exchange [100, 101]. Recent studies have indicated the importance of biomechanical and biochemical changes in the lung microenvironment for IPF, COPD and asthma progression [2, 12, 16, 25, 102–105].

To study the implications of pathological and biomechanical changes due to substrate rigidity as encountered in IPF, on lung fibroblast morphology and migration, Asano et al., seeded HLFs on collagen-I coated PA gels with a stiffness range of 1–50 kPa [106]. On stiffer gels (50 kPa) (which modeled the IPF lung parenchyma), HLFs were more elongated with dendritic extensions, as well as with increased cell projection area, perimeter and aspect ratios whereas, on soft gels (1-2 kPa) fibroblasts were round shaped with no dendritic extensions by the fourth hour and became spindle-shaped by 72 hours  [106]. Cellular migration and cell count were also observed to be higher on the stiffer matrix in comparison to softer matrices, with or without the exogenous supplementation of platelet derived growth factor (PDGF)-BB. Interestingly when FMT was assessed in presence or absence of TGF-β1 stimulations, the expression of *α*-SMA was higher on stiff gels mimicking the IPF microenvironment in comparison to soft gels [106]. In addition to this, Davila et al., compared the gene expression of IPF derived fibroblasts and normal human lung fibroblasts seeded into 3D hydrogels obtained from a decellularized IPF-lung vs. stiff 2D tissue culture plastic dishes [107]. A significant decrease in genes linked with fibroblast activation such as connective tissue growth factor *(CTGF), collagen-1α1 (COL-1A1)*, and *smooth muscle alpha-2 actin* (*ACTA2)* was observed in the fibroblasts seeded in IPF-derived hydrogel as compared to stiffer 2D plastic dishes. Along with the genes associated with fibroblast activation, they also observed a significant reduction in the genes associated with fibroblast proliferation, such as *DNA topoisomerase II alpha (TOP2A)* and *marker of proliferation, Ki-67 (MKI67)* in both IPF derived fibroblasts and normal human lung fibroblasts due to the reduced stiffness of the soft hydrogel in comparison to the stiff culture plate [107]. Again, primary lung fibroblasts from IPF patients and controls embedded in free-floating 3D collagen I and PAA hydrogels mimicking the stiffness of the ‘fibrotic’ and ‘normal’ lung presented with increased *COL1A1* and reduced *matrix metalloproteinase (MMP)-1* expression in response to TGF-β1 stimulation in stiff ‘fibrotic’ compared to the normal gels [108]. Mechanistic experiments revealed that this was mediated by the FAK/Akt pathway. Interestingly, the increased synergistic effect of TGF-β1 and the stiff 3D environment was higher in IPF-derived fibroblasts compared to controls [108]. Further to this, Berhan et al., compared eicosanoid production and signaling pathways in IPF-derived and non-IPF-derived HLFs cultured on soft collagen gels (stiffness range 0.2–0.8 kPa) and 2D monolayer culture on plastic (∼3 GPa) [109]. Here, the expression of cytosolic phospholipase A2 (cPLA2), prostanoid biosynthetic enzyme cyclooxygenase (COX)-2, COX-1, prostaglandin E synthase (PTGES) and subsequently the antifibrogenic lipid mediator prostaglandin E2 (PGE2) were higher in the HLFs cultured on the soft collagen gels, compared to those on 2D plastic substrates [109]. It was found that stiffer matrices stopped the production of PGE2 and mediators in its pathway (COX-2, PTGES) pointing to potentially lower levels of PGE2 in the lungs of IPF patients [109]. The activity of the eicosanoid mediator PGE2 and the different mediators in its pathway have been shown to be antifibrogenic. Hence lower COX-2, COX-1, PTGES and PGE2 due to stiffer matrices adds to fibrotic mechanisms in IPF [109]. This data demonstrates the importance of matrix stiffness in the regulation of antiproliferative and antifibrogenic mediators in IPF pathogenesis.

The biological mechanisms responsible for the transduction of changes in the ECM’s mechanical environment into the nucleus of lung fibroblasts have also been shown to be important for understanding the pathobiology of chronic pulmonary diseases [79, 110–112]. In line with this, Liu et al., developed collagen-I coated PA hydrogels with a stiffness range of 0.4–25 kPa to test how pulmonary fibrosis affects the activity of the mechanotransduction transcription factor, yes-associated protein (YAP) and its ortholog transcriptional coactivator, PDZ-binding motif (TAZ) [110]. Here, a 20–60% significant increase in YAP/TAZ transcripts was observed across all matrix stiffnesses in IPF-derived lung fibroblasts along with a significant increase in YAP/TAZ protein localization in cell nuclei. This showed that YAP/TAZ are central regulators of increased ECM-stiffness-dependent pathologic fibroblast activation in IPF [110]. In agreement with this, Blokland et al., seeded primary lung fibroblasts derived from non-diseased lung tissue onto a 5% w/v bovine serum albumin (BSA) coated GelMa hydrogels with different stiffnesses mimicking a healthy (± 5 kPa stiffness) or COPD -fibrotic (± 15 kPa stiffness) lung tissue to study the mechanosensory response of fibroblasts to pathological changes due to increased ECM stiffness in the lung microenvironment of COPD patients [111]. After experiments, an increased YAP-1 nuclear translocation along with increased decorin protein deposition was found in fibroblasts on stiff hydrogels compared to soft hydrogels [111, 113]. Decorin is an ECM proteoglycan that has been shown to be dysregulated in fibrotic lesions of chronic lung diseases such as COPD [111, 113, 114]. Interestingly, the arrangement of F-actin cytoskeleton in fibroblasts on the COPD-mimicking-fibrotic stiff matrices was tight and bundled whereas on the healthy-mimicking softer matrices, F-actin was a lot more diffused and organized. In addition, there was a significant increase in the total length, area and density of F-actin fibers, along with a higher gene expression of *ACTA2*, *COL*-*1A1* and *fibulin-1* (*FBLN1*) as well as higher FBLN1 protein deposition on stiff matrices compared to soft matrices [111]. Therefore, in all, mechanosensory studies corroborate the findings that exposure of fibroblasts to a microenvironment mimicking increased pathological stiffnesses encountered in chronic lung diseases such as IPF and COPD activates and increases the expression of mechanotransduction transcription factors (YAP, TAZ), fibrotic genes (*ACTA2, COL-1A1, FBLN1*) and proteins that contribute to pathological mechanisms in chronic lung disease.

Further to this, when airway smooth muscle shortening during bronchoconstriction in asthma was mimicked by applying a strain amplitude of 10% to IMR-90 human lung fibroblast cell-lines embedded in 3D collagen I gels, there was increased expression of collagen III, and the activity of MMP-2 and − 9 from fibroblasts [115]. This demonstrated the importance of the mechanical environment in regulating fibroblast phenotype in asthma. Added to this, we have demonstrated a potential novel fibrotic mechanism using primary bronchial fibroblast-embedded collagen I hydrogels that enabled the assessment of the ECM fiber repair phenotype of fibroblasts in the airways [12]. Specifically, we showed that compared to control-derived bronchial fibroblasts, asthma-derived bronchial fibroblasts are less able to contract collagen gels. This was due to their inability to remodel fibrillar collagen in the hydrogels which led to fiber disorganization and fragmentation as demonstrated by multiphoton, second harmonic generation non-linear optical microscopy (SHG-NLOM) [12]. When bronchial biopsies of the same fibroblast donors were examined via SHG-NLOM, it was determined that fibrillar collagen in asthmatic airways were highly disorganized in-line with the collagen-hydrogel data [12]. This demonstrated a possible novel mechanism of fibrosis in asthmatic airways where disorganized and fragmented collagen may stimulate the production of more ECM proteins by fibroblasts [12]. It was found that a lower expression of the proteoglycan decorin in asthma-derived fibroblasts may be a contributing mechanism to this defective fibroblast repair phenotype [12]. To add to the other potential mechanisms that might lead to this defective bronchial fibroblast repair phenotype in asthma, we performed another study and showed that increased concentration of inflammatory mediators in asthmatic airways [116, 117] may also contribute to defective fibroblast repair and collagen fiber disorganization. Here, we found an increased release of the master-regulatory inflammatory cytokine IL-1α and its family member IL-1β from the repairing and differentiating asthma-derived epithelium compared to controls and showed that these cytokines inhibited the ability of primary airway fibroblasts to contract, remodel and organize fibrillar collagen [102, 118]. Mechanistic experiments showed that IL-1 potentially targeted and decreased the expression of the enzyme lysyl oxidase (LOX) and its family members (important enzymes involved in collagen crosslinking) in bronchial fibroblasts to cause the defective repair phenotype [12, 102]. Interestingly, in line with these findings, it has also been shown that lung fibroblasts isolated from distal parenchymal tissue of very severe COPD and emphysema patients are unable to effectively contract collagen I hydrogels [119]. This phenotype was however reversible after the addition of TGF-β or a novel therapeutic tripeptide called GHK indicating a role in potential drug studies [119]. Taken together, these studies point to an emerging role of defective fibroblast repair and disorganization of ECM fibers such as collagen I in lung diseases which has mainly been discovered through collagen hydrogel studies with great potential for future therapeutics.

Altogether, using 3D hydrogel culture models, various studies have demonstrated how defective mechanical properties of the ECM directly influence the abnormal phenotype of fibroblasts and how a defective repair phenotype of fibroblasts is linked to fibrotic mechanisms in chronic lung diseases such as asthma, IPF and COPD (Fig. 1A, B & C). Here it has been shown that in these diseases fibroblasts translate any change in the ECM microenvironment through physical and chemical responses in their phenotype and function such as abnormal migration and cellular elongation, together with dendritic extensions, and actin cytoskeleton organization into rigid bundles, as well as increased proliferation in fibrotic lesions. There is also a defective repair phenotype of airway and parenchymal fibroblasts in asthma and COPD respectively which may lead to fibrillar collagen disorganization and subsequent fibrosis. Interestingly, the inflammatory milieu in diseases such as asthma through the activity of master regulatory cytokines e.g. IL-1 has been implicated in this defective repair phenotype [12, 102, 118, 120–123]. Further, mechanotransduction pathways are also altered as shown in the up-regulated levels of COX-2, cPLA2, PTGES, YAP, TAZ, ACTA2 in fibroblasts which leads to increased expression and deposition of ECM proteins such as *COL-1A1*and *FBLN1* that form fibrotic lesions in IPF, COPD and asthma [12, 102, 109–111, 118, 124].

### 3D hydrogel culture models for therapeutics

Regenerative medicine is continuously evolving and has great potential for improving clinical therapies and patient outcome [121, 123]. Over the last few decades, various advancements involving 3D hydrogel cell culture in the field of tissue engineering have focused on generating hydrogel scaffolds that mimic the ECM for the purpose of in vivo tissue regeneration and repair after tissue destruction [120–123]. Here, recent studies have shown the importance of hydrogel-based culture models as supporting matrix for cell immobilization, drug delivery systems and tissue engineering of complex biomimetic models [125, 126].

In line with this, one of the most common problems faced in lung and thoracic operations are air leaks. An air leak is a clinical phenomenon where an air-containing cavity leaks air into spaces that, under normal circumstances, do not hold air [127–129]. To tackle this phenomenon, Otani and colleagues developed a hydrogel glue, prepared from combining gelatin and poly (L-glutamic acid), to seal lung air leaks [125]. This new hydrogel glue offered a better sealing effect in comparison to previously used fibrin-coated collagen fleeces [130]. In addition to that, it had swellable nanoparticles that has the ability to limit and reduce its removal form the lungs due to macrophagic engulfment. Recently Zhang et al., developed a bioinspired hydrogel with gum arabic, calcium, and pectin infused with basic fibroblast growth factor (bFGF) to stimulate wound healing [131, 132]. Here, in vitro and in vivo results showed that, the non-toxic hydrogel dressing helped in cell proliferation, inflammation, wound re-epithelialization, collagen deposition, and contraction [131]. Similar to this, Hakuba et al., developed and implanted a gelatin-based hydrogel in guinea pig eardrum for a sustained release of bFGF which promoted the closure of perforations and supported the regeneration of fibrous layers in the tympanic membrane [133]. Further, Gao and colleagues used 3D hydrogel cell culturing technology to develop an alternative for docetaxel, a cytotoxic chemotherapeutic agent employed in non-small cell lung (NSCLC) therapy that has adverse hematological effects such as, neutropenia and anemia [125, 126]^,^ [134, 135]. Here, a co-polymer-based hydrogel drug delivery system made up of poly (lactic acid-co-glycolic acid -poly (ethylene glycol) (PLGA-PEG-PLGA), was developed to provide better efficacy for inhibiting tumor growth as the drug could be used for prolonged exposures with minimal side effects [126].

The examples listed above, albeit in different conditions, points to the potential use of lung fibroblast-embedded 3D hydrogels in therapeutic research. These are seen in applications such as integrating multifunctional nanoparticles with pharmaceutical moieties, improving targeted drug delivery (e.g., PLGA-PEG-PLGA), wound dressing, tissue regeneration and building hydrogel-based glues to prevent air leaks in the lungs [125, 126, 131–133].

### Future applications of 3D-Hydrogel (fibroblast) culture models

The human lungs have a complex hierarchical structure and composition with heterogenous mechanical properties that impose dynamic strain conditions on different compartments of the lung tissue during breathing [136, 137]. Chronic pulmonary diseases such as COPD and asthma involve airway thickening and fibrosis while IPF involves lung alveolar/parenchyma remodeling due to, among other things, destruction, disorganization and loss in collagen and elastin fibers, causing substantial mechanodynamic changes and loss of lung elastic recoil [138]. As there is a paucity of studies assessing this, future engineered 3D *invitro* models need to account for the mechanodynamic properties of healthy and diseased lungs. In line with this, we have established lung fibroblast-seeded 3D collagen I hydrogels in the mechanodynamic Flexcell system to which strain at amplitudes and frequencies mimicking the breathing environment can be applied [25]. We here showed that continuous strain led to changes in the morphology of lung fibroblasts that mimic what has been reported in vivo and when fibroblasts were seeded on soft matrices [25, 84]. Further to this we discussed in this review, the use of fibroblast embedded hydrogels to understand mechanisms involved in FMT and EMT. A variation of these processes is endothelial to mesenchymal transition (EndMT), where lung endothelial cells gain mesenchymal markers together with disrupted tight junctions, loss of polarity as well as increased proliferation and migration [139]. The mechanisms involved in EndMT (e.g., increased *Snail*[140] and *S100A4*[141] genes) and how it relates to diseases such as IPF and COPD have been assessed through animal models and 2D cell culture (which are beyond the scope of the current manuscript). Endothelium and fibroblast-embedded 3D-hydrogel (co-culture) studies will further enhance current knowledge on the mechanisms involved in EndMT and aid in therapeutic studies. Finally, through next-generation techniques such as extrusion and digital-light processing bioprinting, 3D hydrogel culture models can be scaled up to mimic the whole lung tissue with multiple cellular components [142]. In line with this, the collagen-elastin-based singular alveolar wall, designed by Dunphy and colleagues can be used as a building block towards engineering whole lung tissue constructs [143]. Further, continuous research and progress in refining the structural components and synthetization methods of the various polymers used to establish 3D hydrogel models have led to the production of more advanced materials such as micro-engineered, supramolecular and nanofiber infused hydrogels, now employed in clinical research and health care to maintain or strengthen the function of load-bearing organs such as the lungs [ 144–146].

## Conclusions

In this review, we provided a summary of the studies that have employed 3D hydrogel culture models to assess fibroblast biology in human lung tissue including, collagen gels, BSA coated GelMa, laminin and fibronectin coated PA gels, cyclic and reversible protein-based hydrogels (Table 1). These studies demonstrate that lung fibroblasts are highly responsive to changes in their ECM microenvironment such as stiffness and adapt their phenotype and function accordingly to match these conditions in health and disease. They also revealed a defective repair phenotype of fibroblasts that leads to ECM disorganization in chronic lung disease. Environments with recurrent mechanical forces or increased stiffness such as those encountered in chronic lung diseases activate mechanotransduction signals to up-regulate fibroblast-dependent ECM activation by increasing fibroblast contractility, proliferation and migration towards fibrotic lesions through EMT and FMT (involving TGF-β1 signaling and increased α-SMA expression). Of interest, the specific mediators involved in lung fibroblast fibrotic responses were shown to include IL-1, COX-2, cPLA2 and PTGES, as well as mechanotransduction transcription factors such as YAP/TAZ which caused increased expression and deposition of fibrotic genes and proteins such as *ACTA2*, α-SMA, *COL-1A1* and *FBLN1*. Hydrogel based scaffold are good models to study how fibroblasts detect and interpret a vast range of ECM’s mechanical cues because they offer easy modification of the physical and chemical properties of established models to effectively mimic different aspects of the ECM microenvironment in health and disease. In addition to the in vitro 3D models, hydrogels are also used in therapeutic applications such as, gelatin and poly L- glutamic acid hydrogel glues and PLGA-PEG-PLGA drug delivery systems with potential for future clinical translation. 3D hydrogel culture technology provides the basis for future work where the incorporation of advanced biomaterials and more cell-types (e.g., immune cells in addition to fibroblasts and epithelial cells) may help develop next-generation lung scaffolding that can be implanted for repair of damaged lung tissue in diseases such as asthma, IPF and COPD.

**Table 1:** Summary of studies assessing lung fibroblast biology using various hydrogel models

3D hydrogel culture models used to access lung fibroblast biology in normal vs. diseased lung				
Type of hydrogel Model	Experimental set up	Mediator(s) Involved	Finding	Ref.
Polyacrylamide hydrogel	IMR-90 cells seeded in a hydrogel (stiffness ranging from 0.3-20 kPa) conjugated with fluorescent beads.	TGF-β1, α-SMA	Lung fibroblasts generated lower force on softer matrices as opposed to greater force on stiffer matrices. Exogenous TGF-β1 treatment promoted α-SMA expression on stiffer matrices in comparison to no effect on softer matrices.	[79]
1D Polyacrylamide hydrogel	CCL-151 lung fibroblasts were seeded in a collagen I functionalized PA hydrogels (stiffness ranging from 0.1-50 kPa)	COX-2, PGE2	Lung fibroblast’s morphology changed from attenuated round with less dendtritic extension cells at lower stiffness to spindle shaped at intermediate stiffness and to spindle shaped with dendrites and parallel swirls at higher stiffness. Stiffness-dependent suppression of COX-2 expression and synthesis of PGE2 during fibrogenesis was also observed in the fibroblasts	[84]
Polyacrylamide hydrogel	Primary alveolar epithelial type II and RLE-6TN were seeded on Fn or Ln coated PA hydrogels (stiffness ranging from 2-32 kPa)	α-SMA, integrin αvβ6/contraction- dependent TGFβ	Primary alveolar epithelial type II and RLE-6TN epithelial cells grown on lower-stiffness (Ln) matrices displayed rounded epithelial morphologies whereas, on higher stiffness (Fn) matrices cells were elongated, contractile and displayed aligned actin filaments like stress fibers. Furthermore, it was observed that low levels of TGF-β caused the epithelial cells to undergo EMT however, upon the removal of TGFβ, cells reverted to an epithelial phenotype.	[78]
Protein-based hydrogel	HLF cells were seeded in a cyclic and reversible hydrogel and observed under reducing and oxidizing conditions (stiffness ranging from 6-20 kPa)	Glutathione as a reducing agent	HLF changed morphology and went from a high cell spread state to a low cell spread state, leading to a significant increase in the cell area, along with a slight decrease in the cell roundness. When conditions were switched back to a reducing state, the opposite was observed, along with clear visibility of actin stress fibers on HLFs indicating better adherence on stiff matrices	[96]
Polyacrylamide gel	HLFs were seeded on collagen-I coated PA gel (stiffness ranging from 1-50 kPa)	*α*-SMA, PDGF-BB, TGF-*β*	On stiffer gels, HLFs were elongated with dendritic extensions, increased cell projection area, perimeter, aspect ratio, cell count and cellular migration whereas, on soft gels fibroblasts presented with less dendritic extensions with or without the exogenous supplementation of PDGF-BB. Furthermore, the expression of *α*-SMA on cells was higher on stiff gels in comparison to soft gels, irrespective of TGF-*β*1.	[106]
Decellularized IPF lung-based hydrogel	An IPF lung was decellularized to produce a hydrogel	*CTGF, COL-1A1, ACTA2, TOP2A, MK167*	In comparison to stiff plastic culture dish, soft hydrogel caused a significant decrease in the genes linked with fibroblast activation and proliferation in both IPF derived and normal lung fibroblasts.	[107]
Free-floating 3D collagen I and PAA hydrogels	Primary lung fibroblasts from IPF patients and controls embedded in free-floating 3D collagen I and PAA hydrogels mimicking the stiffness of the ‘fibrotic’ and ‘normal’ lung	FAK/Akt pathway	Increased *COL-1A1* decrease*d MMP-1* in response to TGF-β. Increased synergistic effect of TGF-β1 and the stiff 3D environment was higher in IPF-derived fibroblasts compared to controls	[108]
Collagen gel	IPF and non-IPF derived cells were seeded on soft collagen gels (stiffness ranging from 0.2–0.8 kPa) and compared to cells seeded on 2D plastic substrates (stiffness around ∼3 Gpa).	PGE2, COX-2, COX-1, cPLA2, PTGES	The expression of COX-2, cPLA2, PGE2 and PTGES were higher in the fibroblasts cultured on the soft collagen gels, compared to those on stiffer 2D plastic substrates.	[109]
Polyacrylamide gel	IPF derived lung fibroblasts were seeded on collagen-I coated PA hydrogel (stiffness ranging from 0.4-25 kPa).	YAP/TAZ transcriptional factors	A 20–60% increase in YAP/TAZ transcripts was observed across all matrix stiffnesses in the IPF-derived lung fibroblasts along with a significant increase in YAP/TAZ protein localization in cell nuclei.	[110]
GelMa hydrogel	Primary HLFs derived from a non-diseased lung tissue were seeded onto a 5% w/v BSA coated GelMa (stiffness ranging from 5-15 kPa).	*ACTA2, collagen-1α1 and FBLN1*, decorin	An increased YAP-1 nuclear translocation along with an increased decorin protein deposition was found in fibroblasts on stiff hydrogels compared to soft hydrogels. The arrangement of F-actin cytoskeleton in fibroblasts on the stiff matrices was tight and bundled in comparison to diffused and organized on softer matrices. There was also significant increase in the total length, area and density of F-actin fibers, along with a higher gene expression of *ACTA2*, *collagen-1α1* and *FBLN1*& FBLN1 protein on stiff matrices compared to soft matrices.	[111]
Collagen I gel	Primary bronchial fibroblasts from asthmatics and non-asthmatics seeded on a free-floating hydrolyzed rat-tail collagen gel I gels	Decorin, lysyl oxidase and its family members and IL-1α/β	Asthma derived airway fibroblasts have low expressions of decorin and are less able to contract collagen gels due to their inability to remodel fibrillar collagen in the hydrogels which led to fiber disorganization and fragmentation. IL-1α and its family member IL-1β targets and decrease the expression levels of the enzyme lysyl oxidase in bronchial fibroblasts to cause fibroblast defective repair phenotype.	[147]
Collagen I gel	Primary human lung fibroblasts were seeded within a 3D collagen gel	TGF-β1/2 and IL-1α	TGF-β1/2 increased the expression of collagen I and α-SMA, however this was inhibited in the presence of IL-1α. IL-1α also inhibited collagen I gel contraction.	[105]
Collagen gels	COPD and emphysema derived lung fibroblasts were cultured on collagen gels	TGF-β, GHK	Lung fibroblasts derived from severe COPD/ emphysema patients were unable to effectively contract collagen hydrogels and repair fibrillar collagen.	[119]
Collagen gels	Lung fibroblasts seeded collagen I gel was established in the mechanodynamic Flexcell system to mimic the breathing environment		Continuous strain leads to changes in the morphology of lung fibroblasts that mimic the observations reported in vivo and when fibroblasts were seeded on soft matrices.	[25]
**3D hydrogel culture models used in therapeutics and future applications**				
Hydrogel Glue	Gelatin combined with poly (L-Glutamic acid)		This hydrogel glue offers better sealing effects for an air leak, which is a common occurrence during lung & thoracic operations.	[125]
Co-polymer drug delivery system	PLGA-PEG-PLGA		An alternative drug delivery system was developed for a cytotoxic chemotherapeutic agent to provide better efficacy with minimal side effects.	[126]
Collagen - elastin based alveolar wall	Collagen and elastin		A singular alveolar wall was built using collagen and elastin that can be used in the future as a building block towards engineering a whole lung tissue construct.	[143]
Hydrogel based wound dressing	bFGF, gum arabic, calcium, pectin		A bioinspired hydrogel based wound dressing was developed to stimulate wound healing.	[131]
Gelatin based hydrogel system	bFGF		A gelatin-based hydrogel system was developed to implant in guinea pigs’ ear for a sustained release of bFGF to generate tympanic membrane and close perforations.	[133]

## Data Availability

NA.

## Abbreviations

ECM: Extracellular matrix
IPF: Idiopathic pulmonary fibrosis
COPD: Chronic obstructive pulmonary disease
1D: One-Dimensional
2D: Two-dimensional
3D: Three-dimensional
GelMa: Gelatin methacrylate
LAP: Lithium phenyl- 2,4,6-trimethylbenzoylphosphinate
MA: Methacrylic anhydride
HA: Hyaluronic acid
CaCl2: Calcium chloride
PEG: Polyethylene glycol
PA: Polyacrylamide
FMT: Fibroblast-to-myofibroblast transition
EMT: Epithelial-to-mesenchymal transition
TGF: Transforming growth factor
αSMA: α-smooth muscle actin
COX: Cyclooxygenase
PGE2: Prostaglandin E2
AEC: Alveolar epithelial cells
Fn: Fibronectin
Ln: Laminin
IF: Immunofluorescence
HLF: Human lung fibroblast
PDGF: Platelet derived growth factor
PTGES: Prostaglandin E synthase
cPLA2: Cytosolic phospholipase A2
YAP: Yes-associated protein
TAZ: Transcriptional coactivator
BSA: Bovine serum albumin
*ACTA2*: Smooth muscle alpha-2 actin
*FBLN1*: Fibulin-1
*COL*-*1A1*: Collagen-1α1
SHG-NLOM: Second harmonic generation non-linear optical microscopy
LOX: Lysyl oxidase
NSCLC: Non-small cell lung
PLGA-PEG-PLGA: Poly (lactic acid-co-glycolic acid -poly (ethylene glycol)
IL: Interleukin
CTGF: Connective tissue growth factor
bFGF: Basic fibroblast growth factor
